# Organizational Practices for the Aging Workforce: Validation of an English Version of the Later Life Workplace Index

**DOI:** 10.1093/geroni/igab046.3031

**Published:** 2021-12-17

**Authors:** Julia Finsel, Anne Wöhrmann, Mo Wang, Max Wilckens, Jürgen Deller

**Affiliations:** 1 Leuphana University Lüneburg, Lüneburg, Niedersachsen, Germany; 2 Federal Institute for Occupational Safety and Health (BAuA), Dortmund, Nordrhein-Westfalen, Germany; 3 University of Florida, Gainesville, Florida, United States; 4 Leuphana University of Lüneburg, Leuphana University of Lüneburg, Niedersachsen, Germany

## Abstract

Due to aging workforces, research on organizational practices for older employees becomes more important for individuals and organizations. However, existing measures for such organizational practices tend to capture the construct with unidimensional scales, use single-item operationalizations, or focus on a specific area. Hence, Wöhrmann, Deller, and Pundt (2018) developed the Later Life Workplace Index (LLWI) to provide a multidimensional framework to measure organizational practices for older employees on nine dimensions, namely organizational climate, leadership, work design, health management, individual development, knowledge management, transition to retirement, continued employment after retirement, and health and retirement coverage. The LLWI has recently been operationalized and validated in Germany (Wilckens, Wöhrmann, Deller, & Wang, 2020). However, to utilize the index beyond German-speaking countries, a validated English version is required. Thus, we aimed to validate an English version of the LLWI using a sample of older U.S. employees (N = 279). Results support the domain level factor structure of the LLWI but show some redundancy among the 80 items for the overall nine domain factor structure. A comparison between the U.S. sample and a German sample (N = 349) confirmed configural and (partial) metric measurement invariance of the English version. Results further supported convergent, discriminant, criterion, as well as incremental validity. Researchers can utilize the new measure to gain a deeper understanding of organizational practices relevant for older employees, while practitioners are able to assess their organizational readiness for an aging workforce. We envision further translation and validation in other languages and cultural contexts.

